# Surgically managed traumatic spinal cord injury in Singapore: a descriptive study across two level one trauma centres

**DOI:** 10.1038/s41394-024-00686-7

**Published:** 2024-11-09

**Authors:** Yong Yao Tan, Lei Jiang, Zhihong Chew, Zhen Yang, Rajashulakshana Rajaram, Mon Hnin Tun, Kappaganthu Venkateshi Prasanna, Li Tat John Chen, Reuben Chee Cheong Soh, Shree Dinesh Kumar

**Affiliations:** 1 Department of Orthopaedic Surgery Changi General Hospital, Singapore, Singapore; 2 Department of Orthopaedic Surgery Singapore General Hospital, Singapore, Singapore; 3 Specialty Nursing Changi General Hospital, Singapore, Singapore; 4 Specialty Nursing Singapore General Hospital, Singapore, Singapore; 5 Health Service Research Changi General Hospital, Singapore, Singapore; 6 Department of Rehabilitation Medicine Changi General Hospital, Singapore, Singapore

**Keywords:** Epidemiology, Risk factors

## Abstract

**Study design:**

Case Series

**Objectives:**

To provide an updated understanding of the incidence of surgically managed Traumatic Spinal Cord Injury (TSCI) in Singapore and to identify factors affecting discharge disposition.

**Setting:**

Patients were identified from two level one trauma centres in Singapore.

**Methods:**

All patients who underwent surgical management for acute TSCI between January 2020 to December 2021 were included. Demographics, injury details, peri-operative condition, hospital length of stay (LOS) and discharge disposition were evaluated. The overall characteristics of TSCI were summarised using descriptive statistics. The difference between discharge destinations was compared using chi-square test or t test. Variables with p values < 0.3 were selected for multivariable analysis.

**Results:**

Forty-four patients were included. Median age was 65. The most common SCI aetiology was fall from standing height or less (54.6%). Accidents involving personal mobility devices, bicycles and motor vehicles made up the next largest group (20.5%). Thirty-nine cases (88.6%) involved the cervical region. There were two cases of inpatient mortality. Twenty-one patients (50%) were discharged home, 21 (50%) were discharged to a community hospital (CH) or nursing home (NH). The median LOS in an acute hospital was 41 days. Multivariable logistic regression analysis revealed that functional independence measure (FIM) score on discharge was an independent factor that influenced discharge disposition (p = 0.037).

**Conclusion:**

A public health focus on falls prevention, the development of geriatric spinal rehabilitation programs, and the consideration of a national registry are recommended for the comprehensive management of TSCI in Singapore.

## Introduction

Traumatic spinal cord injury (TSCI) is a devastating condition with life changing consequences for patients. Globally, the annual incidence rate of TSCI from recent studies has been estimated to range from 38.1 to 49.8 cases per million from Asian studies [1–3], and 26.5–36.6 cases per million from studies conducted in the Western hemisphere [4–6]. While the incidence of TSCI may appear low compared to other conditions, the associated socioeconomic impact is substantial due to the considerable amount, of resources required for both acute and long-term management. It has been estimated that the cost of acute care and inpatient rehabilitation to be up to $130,000 and $180,000 respectively [7]. It has also been estimated that over a one-year period, almost 450,000 DALYs were attributed to TSCI in the United States [8].

In Singapore, a multi-ethnic island state in Southeast Asia boasting an advanced economy and a population of 5.9 million with a median age of 42 years, the last known epidemiological studies on TSCI date back nearly 30 years ago and were limited to a single centre [9, 10]. Considering the profound impact TSCI can have on patients, it is imperative to obtain an updated understanding of TSCI in Singapore. Therefore, the primary aim of this study was to identify the incidence and SCI aetiology of surgically managed TSCI across two tertiary centres that accounts for more than 20% of all acute hospital admissions in Singapore [11]. The secondary aim was to identify factors affecting discharge disposition.

The authors envision this study as a first step towards the establishment of a national registry. This will allow for a systematic capture of comprehensive data on SCI and will enable healthcare professionals to implement more effective preventive measures and enhance the management of patients afflicted with TSCI.

## Methods

This case series involved two tertiary teaching hospitals in Singapore with ethics approval obtained from the Singhealth Centralised Institutional Review Board (IRB). The IRB approval reference number is 2022-030034.

All patients who underwent surgical management for acute TSCI between January 2020 to December 2021 were included in this series. Socio-demographic details collected included age, sex assigned at birth, past medical history (Charlson Comorbidity Index), marital status, pre-morbid living situation and pre-morbid primary occupation. Injury related details studied were SCI aetiology, neurological level of injury (NLI), admission American Spinal Injury Association Impairment Scale (AIS) per the International Standards for Neurological Classification of Spinal Cord Injury guidelines [12], intensive care unit admission and duration on ventilator. Post-operative complications (deep vein thrombosis/pulmonary embolism, myocardial infarction, pneumonia, urinary tract infection, delirium, pressure sore, superficial surgical site infection, unplanned return to theatre, inpatient mortality), hospital length of stay (inclusive of inpatient rehabilitation), discharge disposition (home, community hospital/nursing home), AIS upon discharge and functional independence measure (FIM) score on discharge were also of interest. Variables were referenced from the International Spinal Cord Injury Data Set [13]. The various MOI was referenced from the Singapore National Trauma Registry dataset. The AIS on admission was measured by a spine surgeon prior to patients being offered surgical management whereas the final AIS and FIM score were measured by a rehabilitation medicine physician prior to discharge.

The overall characteristics of spinal cord injury were summarised using descriptive statistics, with continuous variables reported as mean ± standard deviation (SD) or median with interquartile range (IQR), and categorical variables as counts and percentages. The difference between the discharge destinations (home vs community hospital/nursing home) was compared using chi-square test or t test. Variables with p values < 0.3 in univariate analysis were selected for inclusion in a multivariable logistic regression analysis, with odds ratios (OR) and 95% confidence intervals (CI) presented. Stata version 17 (StataCorp LP, College Station, TX) was utilized for all statistical analyses, with statistical significance set at a p value of <0.05.

## Results

### Demographics

From January 2020 to December 2021, there were 44 patients (36 males, 8 females) who underwent surgical management for TSCI across both hospitals. Median age was 65 (IQR 56.3, 71.3). Majority of patients were married (n = 29, 65.9%). Prior to injury, most patients lived with at least one other individual at home (n = 12, 27.3%). There were eight (18.2%) patients who lived alone. Twenty-three (52.3%) patients in this study were also working prior to their injury. The median Charlson Comorbidity Index (CCI) was 1 (IQR 0, 2). The above is summarised in Table 1.

**Table 1:** Demographics of patients.

Variable	All (n = 44) including 2 cases of inpatient mortality	Home (n = 21)	Community hospital/nursing home (n = 21)
**Age (median, IQR)**	65 (56.3, 71.3)	65 (54, 72.25)	65 (54.75, 71)
**Sex Assigned at Birth (n, %)**			
Female	8 (18.18)	6 (28.57)	2 (9.52)
Male	36 (81.82)	15 (71.43)	19 (90.48)
**Marital Status (n, %)**			
Married	29 (65.91)	13 (61.90)	15 (71.43)
Never Married	5 (11.36)	2 (9.52)	3 (14.29)
Divorced	3 (6.82)	1 (4.76)	2 (9.52)
Widowed	3 (6.82)	3 (14.29)	0 (0)
Unknown	4 (9.09)	2 (9.52)	1 (4.76)
**Number of household numbers (n, %)**			
0	8 (18.18)	3 (15.79)	5 (23.81)
1	12 (27.27)	6 (31.58)	5 (23.81)
2	8 (18.18)	3 (15.79)	5 (23.81)
3	4 (9.09)	1 (5.26)	3 (14.29)
>3	9 (20.45)	6 (28.57)	3 (14.29)
Unknown	3 (6.81)	2 (9.52)	0 (0)
**Pre-morbid employment status (n, %)**			
Working	23 (52.27)	13 (61.90)	10 (47.62)
Homemaker, Not working, Retired	14 (31.81)	5 (23.81)	8 (38.10)
Unknown	7 (15.91)	3 (14.29)	3 (14.29)
**CCI (n, %)**			
0	20 (45.45)	9 (42.86)	11 (52.38)
1	9 (20.45)	5 (23.81)	4 (19.05)
2	7 (15.91)	6 (28.57)	1 (4.76)
3	5 (11.36)	0 (0)	3 (14.29)
4	1 (2.27)	1 (4.76)	0 (0)
6	2 (4.55)	0 (0)	2 (9.52)

### Spinal cord injury aetiology

The most common SCI aetiology was fall from standing height or less, with 24 (54.6%) patients suffering a TSCI from that. Accidents involving personal mobility devices, bicycles and motor vehicles made up the next largest group with nine (20.5%) patients sustaining a TSCI from these. Fall from greater than standing height resulted in TSCI in five (11.4%) patients, and six (13.6%) patients had other types of MOI.

### Neurological level of injury and AIS of TSCI

Thirty-nine cases (88.6%) involved the cervical region, three cases (6.8%) involved the thoracic region and two cases (4.6%) involved the lumbar region. There were 12 patients (27.3%) with AIS grade A injury, seven (15.9%) with AIS grade B, 12 with AIS grade C (27.3%) and 13 (29.6%) with AIS grade D injury. Fifteen (34.1%) patients required ICU admission. Among these 15 patients, 13 were TSCI involving the cervical region, two were TSCI involving the lumbar region. The mean ICU LOS was 1.97 days (SD 5.17). The mean number of days on ventilator was 1.3 (SD 4.58). The injury characteristics are summarised in Table 2.

**Table 2:** Injury characteristics.

Variable	All (n = 44) including two cases of inpatient mortality	Home (n = 21)	Community hospital/nursing home (n = 21)
**Neurological level of injury (n, %)**			
Cervical	39 (88.64)	18 (85.71)	19 (90.48)
Thoracic	3 (6.82)	2 (9.52)	1 (4.76)
Lumbar	2 (4.55)	1 (4.76)	1 (4.76)
**AIS on admission (n, %)**			
A	12 (27.27)	2 (9.52)	8 (38.10)
B	7 (15.91)	2 (9.52)	5 (23.81)
C	12 (27.27)	9 (42.86)	3 (14.29)
D	13 (29.55)	8 (38.10)	5 (23.81)
**SCI aetiology (n, %)**			
Fall from standing height or less	24 (54.55)	7 (33.33)	15 (71.43)
Cyclist	6 (13.64)	4 (19.05)	0 (0)
Fall from greater than standing height	5 (11.36)	4 (19.05)	1 (4.76)
Motor vehicle and Motorbike passenger/rider	2 (4.55)	1 (4.76)	1 (4.76)
Personal mobility device rider	1 (2.27)	1 (4.76)	0 (0)
Others	6 (13.64)	4 (19.05)	2 (9.52)
**ICU admission (n, %)**			
No	29 (65.91)	17 (80.95)	12 (57.14)
Yes	15 (34.09)	4 (19.05)	9 (42.86)
**ICU LOS (mean, SD)**	1.97 (5.17)	0.29 (0.72)	3.24 (6.99)
**Days on ventilator (mean, SD)**	1.3 (4.58)	0.09 (0.43)	2.48 (6.38)

### Complications

There were 22 patients with at least one post-operative complication. Urinary tract infection (UTI) was the most common post-operative complication, with 13 patients (29.6%) diagnosed with it. Pneumonia and delirium were the next two most common post-operative complication with seven (15.9%) and five (11.4%) patients diagnosed respectively. There were two cases of inpatient mortality. Both were AIS grade A cervical cord injury requiring ICU admission. The length of stay in ICU were ten days and three days. Complications during acute hospital stay have been summarised in Table 3.

**Table 3:** Complications, function at discharge and length of acute hospital stay.

Variable	All (n = 44) including two cases of inpatient mortality	Home (n = 21)	Community hospital/nursing home (n = 21)
**Complications during inpatient stay (n, %)**			
UTI	13 (29.55)	4 (19.05)	9 (42.86)
Pneumonia	7 (15.91)	1 (4.76)	4 (19.05)
Delirium	5 (11.36)	1 (4.76)	3 (14.29)
MI	2 (4.55)	0 (0)	2 (9.52)
DVT/PE	1 (2.27)	0 (0)	1 (4.76)
Unplanned return to theatre	1 (2.27)	1 (4.76)	0 (0)
**FIM score on discharge (mean, SD)**	66.08 (22.94)	81.86 (15.58)	55.05 (20.89)
**Length of stay (median, IQR)**	41 (26, 64)	38 (25.75, 56)	44.5 (26, 64)

### Factors affecting discharge disposition

Among the 42 patients who were discharged from the acute hospital, 21 patients (50%) were discharged home, 15 (35.7%) were discharged to a community hospital (CH) and six (14.3%) were discharged to a nursing home (NH). Excluding the two patients who demised inpatient, the median LOS in an acute hospital was 41 days (IQR 26, 64). The median LOS was 38 days (IQR 25.8, 56) among those who were discharge home and 44.5 days (IQR 26, 64) among those who were discharged to CH/NH. The mean FIM score on discharge was 66.1 (SD 22.9) for all patients, with those discharged home having a significantly higher FIM score (81.9 vs 55.1, p = 0.001). Refer to Table 3 for a summary of the above.

The multivariable logistic regression analysis revealed that FIM score at discharge was an independent factor that significantly influenced discharge disposition. Specifically, for every one-point increase in FIM score, the odds of discharge to a CH/NH was 0.89 (95% CI 0.81–0.99, p = 0.037). Further information may be found in Table 4.

**Table 4:** Multivariable logistic regression for factors affecting discharge disposition.

Variable	Adjusted odds ratio		
	Estimate	95% CI	p value
**Age**	1.11	0.98–1.25	0.078
**Sex assigned at birth**			
Female	Ref		
Male	0.89	0.04–21.77	0.942
**AIS on admission**			
A	Ref		
B	0.46	0.001–122.21	0.786
C	0.89	0.004–2.26	0.143
D	2.59	0.07–96.53	0.607
**Marital status**			
Married	Ref		
Never married/Divorced/Widowed/unknown	0.56	0.06–4.90	0.597
**Pre-morbid employment status**			
Working	Ref		
Not working/homemaker/retired/unknown	3.45	0.42–28.68	0.252
**Household members**			
<=2	Ref		
>2	0.87	0.14–5.50	0.876
**CCI**			
0	Ref		
1	0.07	0.002–2.76	0.157
>=2	0.02	0.001–2.20	0.100
**FIM score on discharge**	0.89	0.81–0.99	**0.037**

The bold value highlight the variable with *p* < 0.05.

## Discussion

This is the first multicentre study on the incidence of surgically managed TSCI in Singapore. The last known study on the epidemiology of TSCI in Singapore was conducted by Yen et al. [10]. The study included all patients admitted to the Tan Tock Seng Hospital spinal rehabilitation unit from January 1990 to December 1995. It was not specified which patients underwent surgical treatment. The mean age of patients was 39 years old. The most common MOI was a fall from greater than standing height (40.7%) while road traffic accidents take second place (37.2%). Falls from standing height made up 10% of the mechanism of injury for TSCI. The most common level of injury was at the cervical region (53.7%) [10].

There is a notable difference in the demographic profile of TSCI in this study compared to the study by Yen et al. [10]. In the current study, the median age is 65 years, with falls from standing height or less being the predominant SCI aetiology. It is important to highlight that this study spans from January 2020 to December 2021, a period marked by social distancing measures due to the Covid-19 pandemic. During this time, road traffic accidents and workplace accidents reportedly decreased by 50% [14], likely influencing the demographics of patients observed in our study. However, the increase in patient age and the shift in aetiology may also reflect the effects of an aging population, a trend consistent with other studies [1, 6, 15]. Elderly individuals are at a higher risk of falls due to multiple factors such as reduced lower extremity strength, degenerative osteoarthritis of lower limb joints, poorer vision and orthostatic hypotension [16]. Additionally, elderly patients may also have pre-existing cervical spondylosis that can result in a narrower spinal canal [17], increasing risk of cord injury during a fall. The observed reduction in TSCI due to falls from greater than standing height could be attributed to improvements in workplace safety. This is consistent with data from Singapore’s Ministry of Manpower that reported a decrease in both fatal and major workplace injuries over the years [18].

Of note, one patient in our series suffered a TSCI from an accident involving the use of a personal mobility device (PMD). Such devices were not common in the past. In recent years, PMDs have become increasingly popular globally and can have speeds up to 35 km/hr. Accidents involving PMDs can lead to significant injuries, imposing considerable burden on the public health system [19, 20]. Therefore, vigilant monitoring of PMD usage is warranted.

From the results of this study as well as reports from other studies [1, 6, 15], it can be extrapolated that the incidence and prevalence of geriatric SCI patients will likely increase with the years. Addressing this issue requires a two-pronged approach. Firstly, public health initiatives emphasizing falls prevention, especially among the elderly, should be prioritised. Falls pose grave consequences for the elderly population. After a fall, the functional independence and quality of life for an elderly patient can decrease dramatically [21]. Locally, the incidence of unintentional falls among adults aged 60 years and older was 277.7 per 100,000 in 2012, with 85% of geriatric trauma cases in Singapore’s emergency departments being attributed to falls [22, 23]. Considering the complex nature of falls, a multidisciplinary approach is crucial for reducing fall risks in the elderly. In Singapore, falls prevention programs are mainly initiated when clinicians refer patients to physiotherapists or occupational therapists. However, with the rapidly ageing population, attention is being directed towards the community where at risk individuals are identified and recruited into community-based fall prevention programs [24]. Although still in its infancy, early results are promising [25].

Secondly, consideration may be given for the development of geriatric specific SCI rehabilitation programs. For elderly patients who often begin with diminished reserves, the post-operative recovery and rehabilitation process following SCI is inherently protracted and can be more tumultuous compared to younger patients. This is evident from the median LOS in an acute hospital of 41 days reported in our study. Other studies with younger patients had median LOS ranging from 11 to 23 days [26]. A combination of factors such as physiological cognitive decline and the presence of comorbidities constrains the rehabilitation potential of elderly SCI patients. In Yen et al’s study where patients were younger, 3% of them were discharged to a nursing home [10] while 14% of patients in this study were discharged to a nursing home. It is noteworthy that geriatric SCI patients are confronted with a distinctive set of challenges compared to their younger counterparts and may not achieve comparable levels of neurological or functional recovery, a finding that has been reported in the literature [27]. Tailoring programs for geriatric SCI patients may be beneficial.

A notable outcome of this study highlights the factors affecting discharge disposition after TSCI. Multivariable analysis identified a lower FIM score at discharge as an independent predictor of increased likelihood of being discharged to a community hospital or nursing home, consistent with findings from previous studies [28, 29]. This suggests that despite being developed in a Western setting [30], the FIM score is also a valuable tool for rehabilitation physicians to guide discharge planning in our local context. While our study did not investigate the progress of patients in community hospitals, future research could examine the rehabilitation outcomes in such settings to further refine patient care post-TSCI [30, 31].

Finally, this study also underscores the absence of a systematic process for SCI surveillance in Singapore. Establishing a national registry and systematically collecting data will provide valuable insights into both short and long-term outcomes for TSCI patients, aiding in the continual improvement of patient care. One example of such a registry is the Rick Hansen Spinal Cord Injury Registry (RHSCIR), a Canadian-wide database of patients with acute TSCI admitted to trauma centres. Information captured include the pre-hospital, acute and rehabilitation phases, with patients being followed up at one, two, five and then every five years post-injury [31]. Since its establishment in 2004, the RHSCIR was able to provide numerous insights into the management and outcomes of TSCI with subsequent proposal of solutions to further improve the care of patients with TSCI [32]. Setting up a similar registry in Singapore can allow clinicians and allied health professionals to gain invaluable local data for the improvement in care rendered to patients with TSCI. On a global scale, the variables collected in this local registry can be aligned with the International Spinal Cord Injury (InSCI) community survey [33], enabling connections with the international community and contributing to a broader understanding of SCI worldwide.

The authors acknowledge various limitations with this study. Firstly, due to a lack of a systematic surveillance of TSCI, only patients who were surgically managed were captured. This potentially led to a biased cohort evidenced by the low CCI reported in this study. Secondly, this was a retrospective study from January 2020 to December 2021. This was a period where there were social distancing measures implemented due to the Covid-19 pandemic [13] resulting in possibly less work and leisure related injuries. Therefore, the above two factors will result in an underestimate of the incidence of TSCI as well as influence the demographic of patients and distribution of SCI aetiology included in this study.

In conclusion, our study contributes an updated understanding of surgically treated TSCI in Singapore. A public health focus on falls prevention, the development of geriatric spinal rehabilitation programs, and the consideration of a national registry are recommended for the comprehensive management of TSCI in Singapore.

## Data Availability

Data file can be made available upon request to the corresponding author.
